# Plant stress RNA-seq Nexus: a stress-specific transcriptome database in plant cells

**DOI:** 10.1186/s12864-018-5367-5

**Published:** 2018-12-27

**Authors:** Jian-Rong Li, Chun-Chi Liu, Chuan-Hu Sun, Yu-Ting Chen

**Affiliations:** 1 Program in Medical Biotechnology, National Chung Hsing University, 145 Xingda Rd., South Dist, Taichung City, 402 Taiwan; 2 Advanced Plant Biotechnology Center, National Chung Hsing University, 145 Xingda Rd., South Dist, Taichung City, 402 Taiwan; 3 Institute of Genomics and Bioinformatics, National Chung Hsing University, 145 Xingda Rd., South Dist, Taichung City, 402 Taiwan

**Keywords:** RNA-Seq, Plant stress, Database, Differential expression

## Abstract

**Background:**

Abiotic and biotic stresses severely affect the growth and reproduction of plants and crops. Determining the critical molecular mechanisms and cellular processes in response to stresses will provide biological insight for addressing both climate change and food crises. RNA sequencing (RNA-Seq) is a revolutionary tool that has been used extensively in plant stress research. However, no existing large-scale RNA-Seq database has been designed to provide information on the stress-specific differentially expressed transcripts that occur across diverse plant species and various stresses.

**Results:**

We have constructed a comprehensive database, the plant stress RNA-Seq nexus (PSRN), which includes 12 plant species, 26 plant-stress RNA-Seq datasets, and 937 samples. All samples are assigned to 133 stress-specific subsets, which are constructed into 254 subset pairs, a comparison between selected two subsets, for stress-specific differentially expressed transcript identification.

**Conclusions:**

PSRN is an open resource for intuitive data exploration, providing expression profiles of coding-transcript/lncRNA and identifying which transcripts are differentially expressed between different stress-specific subsets, in order to support researchers generating new biological insights and hypotheses in molecular breeding or evolution. PSRN is freely available at http://syslab5.nchu.edu.tw/PSRN.

**Electronic supplementary material:**

The online version of this article (10.1186/s12864-018-5367-5) contains supplementary material, which is available to authorized users.

## Background

Abiotic and biotic stresses are the most harmful factors affecting the growth and productivity of crops and leading to food crisis worldwide. Drought, salinity, heat, cold, chilling, freezing, nutrient, high light intensity, ozone (O_3_) and anaerobic stresses are the main abiotic stresses [1]. Along with abiotic stresses, plants also have to face biotic stresses caused by pathogens (including bacteria, fungi, viruses, and nematodes) and herbivorous pests [2]. Recent studies showed that abiotic stresses, such as rising temperatures, can facilitate the spread of pathogens and weaken the defense mechanisms of plants [3, 4]. To improve the resistance to stress, several cultivars and mutant lines have been developed [5, 6]. In response to stress, plant physiology and transcriptomes undergo changes in alarm, resistance, exhaustion, and regeneration phases [7, 8]. Since different tissues and developmental stages present different resistances to stress [9, 10], transcriptome profiling of different tissues, strains, and developmental stages under various environmental stress conditions could provide insights into the molecular mechanisms as how plants respond to stress.

RNA sequencing (RNA-Seq) [11] is a revolutionary tool that can quantify gene/isoform expression levels at a higher resolution than microarray technology and provide coding-transcript profiling as well as long noncoding RNA (lncRNA) profiling. RNA-Seq has been used extensively in plant research. Recently, global transcriptome profiling analysis using RNA-Seq has been reported to identify differentially expressed lncRNAs, coding genes and alternatively spliced isoforms in response to environmental stresses, such as salt, heat, cold, drought, light, ozone, excessive boron, and pathogen infection [12–14]. Meanwhile, there now exist several publicly available databases built for plant stress, such as RiceSRTFDB [15], STIFDB2 [16], PASmiR [17], QlicRice [18], PhytAMP [19], and PSPDB [20]. Among them, RiceSRTFDB and STIFDB2 are curated with microarray data and focused on transcription factors. The development of PASmiR was based on abiotic stress responsive miRNA. PSPDB was constructed with biotic and abiotic stress protein annotations. Only the PRGdb 2.0 database collected RNA-Seq data, but that data was limited to resistance genes (R-genes). In addition, several data portals contain a vast amount of plant RNA-Seq data, such as the National Center for Biotechnology Information (NCBI) Gene Expression Omnibus (GEO) [21] and the Sequence Read Archive (SRA) [22]. However, these data portals mainly serve as raw biological data archives. Therefore, a large-scale stress-specific RNA-Seq database that can provide comprehensively visualized transcriptome expression profiles and statistical analysis for differential expression has not been reported for plants. In the postgenomic era, RNA-Seq provides a global transcriptome profile, which could cover lncRNAs, coding genes and their alternatively spliced isoforms in response, and helps plant biologists to expand new insights into molecular mechanisms and responses to biotic and abiotic events. Thus, we developed an extensive genome-wide plant stress RNA-Seq database.

**Fig. 1:**
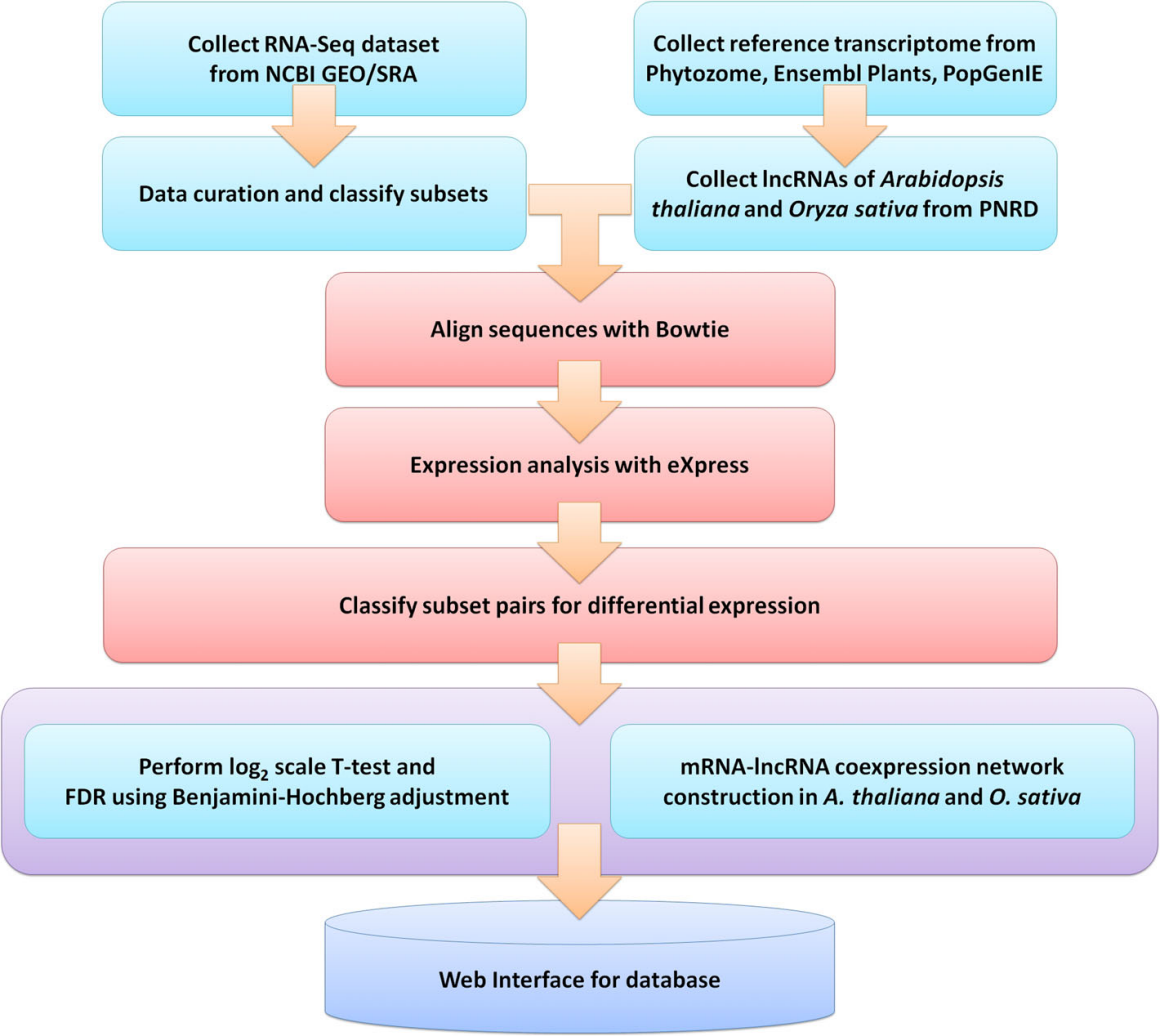
The framework of the database construction in PSRN. The plant stress RNA-Seq datasets were collected from NCBI GEO and SRA, and then all samples were classified into stress-specific subsets for each dataset. In the RNA-Seq data processing, Bowtie2 and eXpress were used to calculate transcript expressions of each RNA-Seq dataset with references collected from Phytozome, Ensembl Plants, and PopGenIE. Finally, we calculated the log2 scale T-test and FDR between two subsets belonging to the same dataset and then constructed the user interface for PSRN

In this study, we constructed the first large-scale plant stress RNA-Seq database, the *plant stress RNA-Seq nexus (PSRN)*, that achieves the following unprecedented features: (i) large-scale and comprehensive data archives and analyses, including coding-transcript profiling and lncRNA profiling, (ii) phenotype-oriented data organization and searching, and (iii) the visualization of expression profiles, as well as differential expression. PSRN was developed with the goal of collecting, processing, analyzing and visualizing publicly available plant RNA-Seq data. Figure 1 presents the framework of PSRN database construction. It resulted in 12 plant species and 26 plant-stress RNA-Seq datasets, including 133 stress-specific subsets and 937 samples (Additional file 1: Table S1). Each subset is a group of plant RNA-Seq samples associated with a specific stress phenotype or genotype. In addition, PSRN provides a user-friendly interface to efficiently organize and visualize the expression profiles of the differential expressed transcripts for any pair of stress-specific subsets (Additional file 2: Table S2). PSRN is freely available at *http://syslab5.nchu.edu.tw/PSRN*.

### Construction and content

#### Plant stress RNA-Seq dataset collection

The annotation for the RNA-Seq datasets regarding plant stress was collected from NCBI GEO [21], and RNA-Seq reads were downloaded from the SRA [22]. Each dataset has several stress-specific subsets that contain a group of RNA-Seq samples of plants treated with a specific stress condition, e.g., salt, heat cold, drought, and light. We manually created subsets and then systematically assigned samples to subsets according to the descriptions of the datasets and samples. For expression analysis, the reference sequences and annotations of the protein-coding transcriptome were collected from Phytozome v11 [23], Ensembl Plants [24], and PopGenIE [25]. Moreover, the long noncoding RNAs (lncRNAs) of *Arabidopsis thaliana* and *Oryza sativa* were obtained from plant non-coding RNA database (PNRD) [26] for the plant lncRNAs expression profile analysis. We only retained the datasets that had a reference transcriptome for subsequent RNA-Seq analysis. In addition to transcriptome annotations collected from the abovementioned databases, the transcriptome of each species was employed for homology searches against plant sequences retrieved from the NCBI RefSeq database [27] using the NCBI BLAST+ toolkit [28], version 2.3.0, with an E-value threshold of 1E-3. Furthermore, KAAS (http://www.genome.jp/tools/kaas/) [29] was used for homology searches of KEGG and pathway annotation [30] of each plant transcriptome.

#### Stress-specific differentially expressed transcripts

To estimate the expression profile of each sample, Bowtie [31], version 1.1.2, was used to build the indexer of the reference sequence and align RNA-Seq reads to the reference transcriptome with the indexer. Samples with at least 1 million raw reads and over 15% of reads mapped to the reference transcriptome were retained for subsequent analysis to ensure enough depth of the sequencing coverage [32]. After alignment, transcript abundances of each sample were estimated using eXpress [33], version 1.5.1, with the expression quantification unit ‘fragments per kilobase of transcript per million mapped reads (FPKM) [34]. To identify stress-specific, differentially expressed, transcripts in each dataset, we selected the subsets that included at least three samples to fulfill the significance test criteria, and then converted the expression FPKM value to a log_2_ scale with an added pseudo-count and performed a *t*-test between two subsets without overlapping samples. To take the type-I error rate into account, we simultaneously used the Benjamini-Hochberg procedure [35] to calculate the false discovery rate (FDR). This resulted in 259 stress-specific subset pairs with differentially expressed transcripts (*P*-value < 0.01).

#### Protein-coding RNA-lncRNA coexpression networks

To investigate the potential stress-specific biological functions of lncRNAs in plant cells, we constructed protein-coding RNA and lncRNA coexpression networks from *Arabidopsis thaliana* and *Oryza sativa* data. We selected coding transcripts and lncRNAs with a relatively high expression variance (standard deviation > 0.3 and (standard deviation/mean) > 0.5) within a subset and then calculated Pearson’s correlation coefficient between every pair of selected coding RNA and lncRNA expression profiles for a given subset pair. To assess the significance of these connections, the Pearson’s correlation coefficient was transferred into the *P*-value and the significant correlations (*P*-value <1E-6) between protein-coding RNAs and lncRNAs were collected for network construction. Cytoscape [36] was used to demonstrate the protein-coding RNA-lncRNA coexpression networks in the web interface of PSRN.

## Utility and discussion

The PSRN database includes 12 plant species: *Arabidopsis thaliana*, *Chlamydomonas reinhardtii*, *Glycine max*, *Manihot esculenta*, *Oryza sativa* indica, *Oryza sativa* Japonica, *Panicum virgatum*, *Populus tremuloides*, *Solanum lycopersicum*, *Sorghum bicolor*, *Triticum aestivum*, and *Vitis vinifera*, which contain 26 RNA-Seq datasets and 937 samples. All samples were classified and assigned to 133 stress-specific subsets, which were constructed into 254 subset pairs to describe stress-specific differentially expressed (DE) transcripts from a systematic RNA-Seq analysis. Considering the variants exiting in different analyses, we just compared the expression profile in the same database. We have also checked the consistency between PSRN and the individual analysis in each paper. According to the information of individual papers, we used transcript ID or gene name to find the corresponding expression profile. Most of them displayed similar patterns between PSRN and the individual analyses in different papers.

### Web interface

**Fig. 2:**
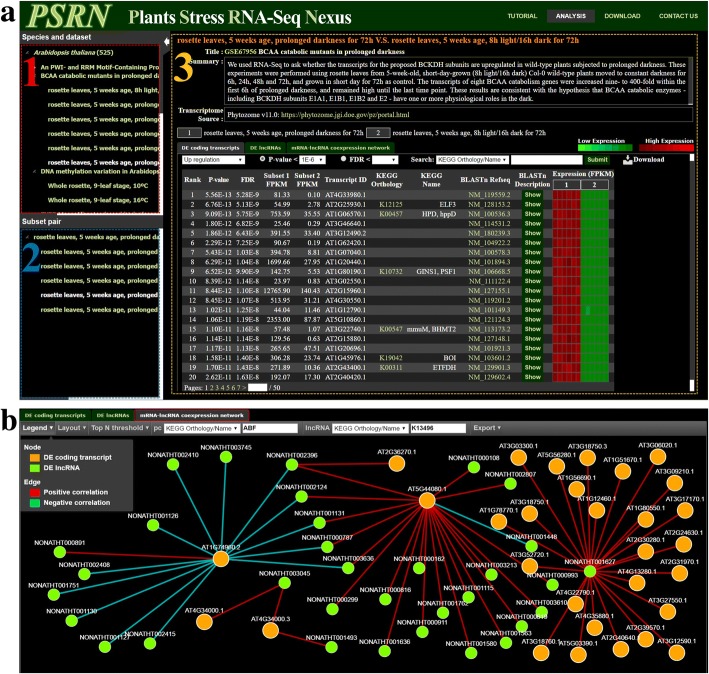
Screenshots of the web interface of PSRN. (**a**) The PSRN user interface consists of three major panels as follows: **(1) Species and subset panel**: users can browse plant species and phenotype-specific subsets. **(2) Subset-pair panel**: given the phenotype-specific subset, users can select an associated subset pair. **(3) Expression profile panel**: subset-pair information and differentially expressed protein-coding transcripts are shown in the panel and sorted by significance level. When users use Search in the expression panel, they can input a transcript ID, KEGG Orthology/name, or RefSeq ID into the autocomplete field that allows for quickly searching and selecting the partially matched terms. Furthermore, by entering more characters, it will filter down the list to better matches. At last, PSRN generates the expression profiles of all isoforms in the search results. In addition, users can download the result generated from PSRN as a file with PDF format. Moreover, users can select a *P* value or FDR threshold and regulation type to identify significantly differentially expressed transcripts. If a user clicks “DE lncRNAs” in *Arabidopsis thaliana* and *Oryza sativa*, differentially expressed lncRNA transcripts are replaced with protein-coding isoforms. (**b**) The lncRNA regulatory network function in *A. thaliana* and *O. sativa* presents the regulatory network according to the correlation of expression between lncRNAs and protein-coding transcripts

PSRN provides a user-friendly web interface that integrates large-scale stress-specific RNA-Seq datasets of plants. Three main functional units, analysis, tutorial, and download, were included in PSRN. The tutorial unit provides brief instructions about how to use PSRN. From the download unit, users can retrieve all the differential expression analysis results of all datasets in PSRN. Here, we describe the analysis unit, which is the main function unit of the PSRN database. As shown in Fig. 2a, the analysis function provides a tree structure in the species and subset panel, which facilitates searching and browsing stress-specific subsets. When users select a subset of interest, the associated subset pairs are subsequently listed in the subset-pair panel. When users select a subset pair, the web server shows the detailed information of the DE transcripts of the subset pair, as well as the detailed description of the dataset and subsets, into the right main panel. In the right main panel, there are three subpanel tabs as follows:

(i) *DE coding transcripts:* Given a subset pair, PSRN constructs a heat map to visualize the expression profiles of all DE protein-coding transcripts, sorted by the significance level (*P*-value) between two subsets. The interface displays the rank, the *P*-value, the FDR, the average expression values in the subset, the transcript ID and the homology annotation for each transcript, such as KEGG and RefSeq annotations. Whenever user clicks transcript ID, the PSRN will show details of its expression in all subsets across all datasets belonging to the same species. In this panel, users can filter DE transcripts based on their *P*-value or FDR threshold and choose to present the sorted transcripts according to whether they are up- or downregulated. Additionally, the search function on the expression profiles allows users to investigate all transcripts associated with the given transcript ID, KEGG Orthology number/Name, or RefSeq annotation, and an autocomplete function provides suggestions for search field as the user types, quickly searching and displaying partially matched terms. The search function also allows users to investigate the expression profiles of multiple genes at one time. To do so, users simply type their transcript IDs, KEGG Orthology numbers/Names, or RefSeq annotations, and separate them by comma.
(ii) *DE lncRNAs*: The DE lncRNAs tab panel is only provided for *Arabidopsis thaliana* and *Oryza sativa* (both in indica group and japonica group). All functions of this panel are similar to the DE coding transcript panel but visualize the expression profiles of DE lncRNAs sorted by the *P*-value.
(iii) *Protein-coding RNA-lncRNA coexpression network*: As shown in ​Fig. 2b, PSRN visualizes the coding RNA-lncRNA coexpression network to depict the significant correlations between coding transcripts and lncRNAs. Given the subset pair, users can input a transcript ID/KEGG Orthology/Name/RefSeq annotation of a coding transcript and lncRNA into the autocomplete field. Furthermore, users can select the top 10/15/20/25 most significant connections to show for the given coding transcripts and/or the given lncRNA. Similar to the DE lncRNA panel, this panel is only constructed for *Arabidopsis thaliana* and *Oryza sativa* groups.

### Case study

To demonstrate the biological functionality of PSRN, we used cold-related transcripts and HAB1 isoforms of *Arabidopsis thaliana* as examples.

**Fig. 3:**
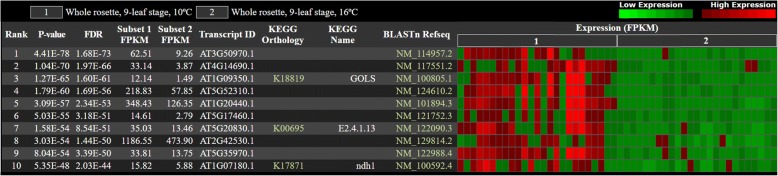
The expression of cold-related transcripts in *Arabidopsis thaliana* treated with different temperature conditions. The top 5 significantly upregulated transcripts in the 10 °C subset are AT3G50970.1, AT4G14690.1, AT1G09350.1, AT5G52310.1, and AT1G20440.1 (*P*-value = 4.41E-78 ~ 3.09E-57; FDR = 1.68E-73 ~ 2.34E-53). According to the TAIR database, AT3G50970.1, AT5G52310.1, and AT1G20440.1 are Low Temperature-Induced 30 (LTI30, XERO2), Low-Temperature-Induced 78 (LTI78, COR78), and Cold-Regulated 47 (COR47, ATCOR47), respectively. In addition, AT4G14690.1 is Early Light-Inducible Protein 2 (ELIP2), and AT1G09350.1 is Galactinol Synthase 3 (GOLS3, ATGOLS3). Limited by the layout width, only parts of the expression profiles are shown

**Fig. 4:**
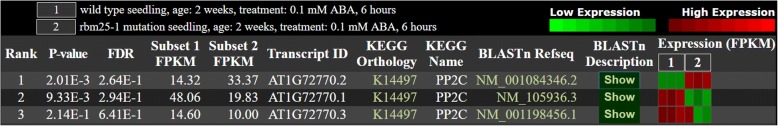
The expression of HAB1 isoforms of the seedlings treated with ABA in *Arabidopsis thaliana*. The group A PP2C (protein phosphatases 2C) HAB1 gene has three transcript isoforms: HAB1.1 (AT1G72770.1), HAB1.2 (AT1G72770.2), and HAB1.3 (AT1G72770.3). Based on the *P*-value calculated by PSRN, the HAB1 isoform AT1G72770.1 is significantly downregulated (*P*-value = 9.33E-03) in the rbm25–1 mutation seedlings when compared with the wild type, while AT1G72770.2 is significantly upregulated (*P*-value = 2.01E-3) in rbm25–1 mutant seedlings

(i) In the analysis results for the GSE54680 dataset, the top 5 significant upregulated transcripts in 10 °C subset are AT3G50970.1 (Low Temperature-Induced 30, LTI30, XERO2), AT4G14690.1 (Early Light-Inducible Protein 2, ELIP2), AT1G09350.1 (Galactinol Synthase 3, GOLS3, ATGOLS3), AT5G52310.1 (Low- Temperature-Induced 78, LTI78, COR78), and AT1G20440.1 (Cold-Regulated 47, COR47, ATCOR47) (illustrated in Fig. 3). LTI30 is a late embryogenesis abundant protein (LEA)/dehydrin that can bind to and protect membranes to enhance freezing stress resistance in *Arabidopsis* [37, 38]. ELIP 2, a thylakoid protein, was induced not only by the cold but also by UVB and high light for protective photoinhibition under stresses [39]. Raffinose family oligosaccharides (RFO) were identified as being involved in tolerance to drought, high salinity and cold stresses. GOLS catalyzes the first step in the biosynthesis of RFO. In *Arabidopsis*, AtGOLS1 and AtGOLS12 were induced by drought and high-salinity stresses; in contrast, AtGOLS3 was induced by cold stress only [40]. COR78 [41] and COR47 [42] are well-known cold-regulated (COR) genes, and high expression levels of COR47 and COR78 in response to cold acclimation has been reported [5]. The abovementioned results are consistent with previous studies.
(ii) In the analysis results for the GSE66737 dataset, we used the HAB1 gene, a group A protein phosphatase 2C (PP2C), to demonstrate the biological importance of the level of expression of isoforms provided in PSRN (illustrated in Fig. 4). HAB1 is a coreceptor of abscisic acid (ABA) and an important negative regulator of ABA signaling. In *Arabidopsis*, the HAB1 gene has three alternatively spliced isoforms: HAB1.1 (AT1G72770.1), HAB1.2 (AT1G72770.2), and HAB1.3 (AT1G72770.3). HAB1.1 interacts with the Open Stomata 1 (OST1), inhibiting its kinase activity, which switches the ABA signaling off. In contrast, HAB1.2 encodes a nonfunctional truncated protein, thereby keeping the ABA signaling on. Thus, accurate regulation of the HAB1.1 to HAB1.2 ratio is important for the fine-tuning of ABA signaling and plant adaptation to stress. An RNA-binding motif (RBM)-containing protein RBM 25 is known as a critical key regulator of HAB1 isoforms through its binding to the last intron of HAB1 pre-mRNA in *Arabidopsis* [43]. A loss-of-function mutation in RBM25, rbm25–1, resulted in an increase in the HAB1.2:HAB1.1 ratio and ABA-hypersensitive phenotypes [44]. As shown in Fig. 4, HAB1.1 (At1g72770.1) is significantly downregulated while HAB1.2 (At1g72770.2) is significantly upregulated in rbm25–1 mutation seedlings, which is consistent with previous reports.

### Future developments

Despite numerous RNA-Seq datasets being collected at the beginning of the study, only a minority of them were retained for PSRN construction. Most of the discarded datasets were excluded because of the insufficient number of samples suitable for the criteria of subset creation, and the rest were excluded because of a lack of a reference sequence. To solve the latter problem, we will continue to collect references, annotations, and RNA-Seq datasets to expand the PSRN database and keep it up to date. The next updated version will include: (1) the new plant stress RNA-Seq datasets and the reference transcriptomes collection, data curation, and KEGG and Refseq annotations, (2) performing the expression profile analysis pipeline of RNA-Seq datasets. The DESeq will be performed in this update, and the results of both t-test and DESeq will be reported in the database.

## Conclusions

In this study, we collected, processed, analyzed and visualized all publicly available plant stress RNA-Seq data from NCBI GEO and SRA to construct the PSRN database. Although a few databases have previously been developed for plant stress, PSRN is the first comprehensive database using RNA-Seq data to obtain complete transcriptome profiles and differentially expressed protein-coding transcripts and lncRNAs for various plant species and stress types. Moreover, the systematic and user-friendly web interface can assist researchers in accessing the information efficiently. We hope that PSRN may provide a new resource that can facilitate biologists gaining better biological insights into plant responses to stress with a high resolution of transcript isoforms levels.

## Additional files

Additional file 1: **Table S1.** The list of 133 subsets in 26 datasets of PSRN. (XLSX 15 kb)

Additional file 2: **Table S2.** The stress treatment information and web link of subset pairs in PSRN. (XLSX 37 kb)

## Abbreviations

FDR: false discovery rate
FPKM: fragments per kilobase of transcript per million mapped reads
GEO: Gene Expression Omnibus
KEGG: Kyoto Encyclopedia of Genes and Genomes
lncRNA: long noncoding RNA
NCBI: National Center for Biotechnology Information
PNRD: a plant non-coding RNA database
RNA-Seq: RNA sequencing
SRA: Sequence Read Archive
